# Treatment and prevention of HIV/AIDS: Unfinished business

**DOI:** 10.1371/journal.pmed.1004806

**Published:** 2025-12-01

**Authors:** Anthony S. Fauci, Gregory K. Folkers

**Affiliations:** 1 School of Medicine and McCourt School of Public Policy, Georgetown University, Washington, DC, United States of America; 2 Independent, Washington, DC, United States of America

## Abstract

In recognition of World AIDS Day 2025, this Perspective by Gregory Folkers and Anthony Fauci reflects on the progress made in antiretroviral treatments and prevention of HIV/AIDS, highlighting promising therapeutic developments and looking ahead to what is needed to end the AIDS epidemic.

## Teaser

Since the inception of World AIDS Day in 1988, advances with antiretroviral drugs have revolutionized the landscape of HIV/AIDS treatment and prevention. In 2025, we reflect on progress made, highlight promising therapeutic developments, and look ahead to what is needed to end the AIDS epidemic.

**Fig 1:**
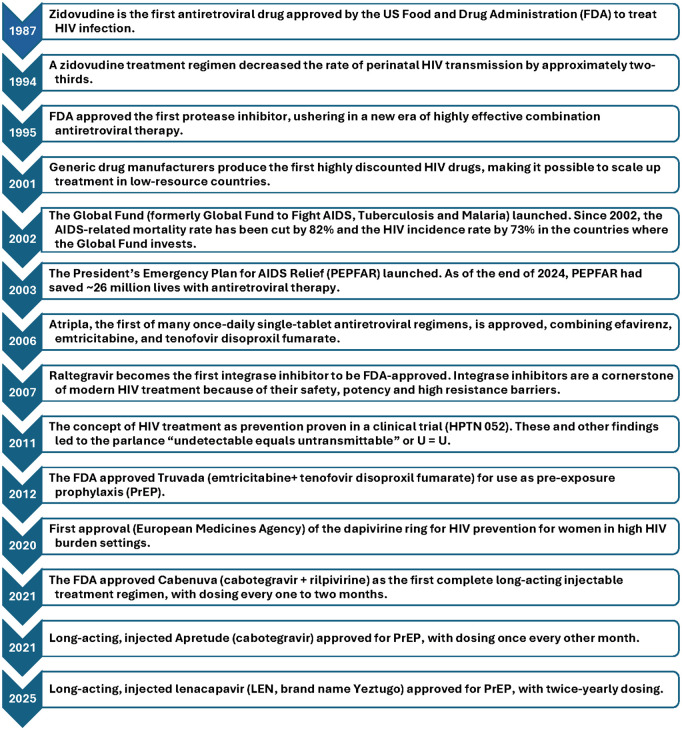
Selected milestones in antiretroviral-based HIV treatment and prevention.

World AIDS Day was first commemorated on 1st December 1988, a time when tools for HIV treatment and prevention were limited, and HIV incidence, prevalence, and mortality in the United States (US) and globally were rapidly increasing. Thirty-seven years later, the number of people living with HIV globally exceeds 40 million, with 1.3 million new infections and 660,000 deaths in 2024 alone [1]. Fortunately, advances in HIV science, driven by robust funding and the efforts of a broad coalition of multiple communities, has provided an array of effective tools to prevent and treat HIV, especially interventions based on antiretroviral drugs (see Fig 1).

Beginning in the mid-1990s, multidrug antiretroviral regimens transformed the prognoses for people with HIV from dire to excellent. Antiretroviral therapy can reduce virus in the blood to undetectable levels, preserving the health of the person and preventing the transmission of the virus to sexual partners. The latter effect forms the basis for the concept of “undetectable equals untransmittable” or U=U. Over the years, progressively more potent, better-tolerated antiretroviral drug combinations, often taken as one daily pill, have further improved HIV treatment [2].

In the realm of HIV prevention, pre-exposure prophylaxis (PrEP) with two oral antiretroviral drugs (or injected cabotegravir) taken before a person is exposed to HIV can be more than 99% effective in preventing sexual acquisition of HIV. The dapivirine vaginal ring is available as an additional prevention choice for women at substantial risk of HIV infection [1]. Most recently, the long-acting HIV capsid inhibitor lenacapavir (LEN), given subcutaneously once every 6 months, has shown near-perfect efficacy as PrEP in phase 3 clinical trials. The efficacy of injectable LEN was 100% among women and adolescent girls in the PURPOSE 1 trial (*n* = 5,338) [3] and 96% among men and gender-diverse people in PURPOSE 2 (*n* = 3,265) [4]. LEN has been generally well-tolerated and perceived as highly acceptable, with users citing its convenience and potential for discreet use [5]. Mathematical models suggest that LEN could substantially reduce new HIV infections, especially where uptake and adherence are optimized. LEN is approved by the US Food and Drug Administration, the European Medicines Agency, and regulatory authorities in South Africa and Zambia. It is recommended by the World Health Organization [5] and the US Centers for Disease Control and Prevention [6] as part of combination prevention approaches. Because of its infrequent dosing, LEN has been called the “next best thing to a vaccine” for preventing HIV infection and holds great promise for improving PrEP adherence. With current oral PrEP regimens, less than half of people are still taking the drugs a year after beginning prophylaxis [6].

While these groundbreaking developments have provided us with powerful therapeutics to fight HIV, equitable access to these tools has been a barrier. However, over the past two decades, we have learned that antiretroviral-based HIV treatment and prevention interventions can be successfully implemented and scaled-up in low- and middle-income countries (LMICs). Notably, programs such as the Global Fund and the President’s Emergency Plan for HIV Relief (PEPFAR) have saved millions of lives with antiretroviral drugs and other HIV-related services (see Fig 1). These programs have greatly expanded HIV treatment and wrap-around services in LMICs and have built workforce, laboratory, and clinical capacity, using sophisticated monitoring and evaluating systems to assess and improve results and ensure accountability [1,7].

As a result of these and other programs, since 2010 antiretroviral drugs have reached 77% of people with HIV globally, and annual new HIV infections and HIV-related deaths have declined by 54% and 40%, respectively [1]. At least seven countries—Botswana, Eswatini, Lesotho, Namibia, Rwanda, Zambia, and Zimbabwe—have reached “95-95-95” targets: That is, 95% of people living with HIV know their HIV status; 95% of people who know their status are receiving antiretroviral therapy; and 95% of people on treatment achieve viral suppression [1]. PrEP has also begun to reach poorer countries, albeit haltingly. In 2024, 2.5 million people received PrEP through PEPFAR [1].

Progress with antiretroviral-based treatment and prevention prompts us to consider an aspirational goal. If we could identify with widespread and routine testing every person with HIV in the world and treated them with antiretroviral drugs to reduce their viral load below detectable levels, and put all at-risk people on effective PrEP regimens, we could achieve a world where new HIV infections and deaths from advanced HIV/AIDS were very rare. Although this would be logistically difficult due to the global disparities in access to healthcare, countries that have achieved 95-95-95 status due to the Global Fund, PEPFAR, and in-country programs suggest that this goal is not as far-fetched as it might seem.

Progress in curbing the global HIV pandemic has been slowed by pauses in US foreign development assistance and stop-work orders on existing grants and contracts at PEPFAR and other programs [8]. Detailed information on the current status of PEPFAR programs has not been made public. However, outside observers have reported that thousands of staff have been laid off and facilities closed, and that stock-outs of antiretroviral drugs for treatment and PrEP, viral load tests, and other products have occurred [8]. The termination of clinical services likely has resulted in the illness and death of thousands of people with HIV. Modeling studies suggest that millions of additional HIV infections and deaths could occur if withheld funding is not reconstituted and expanded [8].

The future of PEPFAR remains uncertain. A recent US State Department global health strategy suggests that future PEPFAR activities will focus mostly on commodity purchases such as diagnostic and drugs, and salaries for frontline and healthcare workers serving patients directly, and that other services will transition to in-country providers [9]. In September 2025, PEPFAR and the Global Fund committed to reaching 2 million people with LEN in 8–12 (thus-far unnamed) countries with high burdens of HIV, with an emphasis on preventing mother-to-child transmission [10]. This effort is a good start, but to slow the momentum of the HIV/AIDS pandemic, it will be crucial that LEN reach many more people who need it. This is especially true for adolescent girls and young women (aged 15–24 years) who accounted for 210,000 new infections 2024, and so-called “key populations” (men who have sex with men; sex workers; people who inject drugs; transgender people; incarcerated individuals) and their sexual partners, who account for >50% of all new infections [1]. UNAIDS has set an aspirational goal for 20 million people in high-need populations to have access to long-acting HIV prevention medicines, including LEN, by 2030 [1].

Current manufacturing capacity, together with further procurement investments, could allow LEN to reach 5 million people over the next 3 years [11]. Recent agreements to produce generic versions of LEN for $40 per person per year promise much greater future access [12]. With additional generic manufacturers of LEN, greater price reductions, and well-designed and funded programs for PrEP distribution, it might be possible to reach >7 million people with LEN by 2030 [11].

After nearly four decades of progress with antiretroviral-based treatment and prevention for HIV, we now have truly transformational tools to end the HIV/AIDS pandemic as a major global health threat. History will judge us harshly should we squander this opportunity. The time is now to advocate for the US Congress to renew funding of the Global Fund and PEPFAR at robust levels to scale up LEN and other interventions. However, these programs cannot assume the entire burden of support alone. As more countries take control of their own HIV responses, innovative, talented, and creative people in numerous sectors must make ambitious commitments to procuring and delivering antiretroviral-based interventions. National governments, international agencies, donors, community groups, drug manufacturers, researchers, and implementers all have important roles to play in developing programs and processes for scaling up life-saving HIV treatments and prevention so that they reach all in need. Only with such a multi-pronged effort will we end the HIV/AIDS pandemic.

## Abbreviations

LEN: lenacapavir
LMICs: low- and middle-income countries
PrEP: pre-exposure prophylaxis
PEPFAR: President’s Emergency Plan for AIDS Relief
